# The Variable Hinge Region of Novel PKCs Determines Localization to Distinct Regions of the Immunological Synapse

**DOI:** 10.1371/journal.pone.0095531

**Published:** 2014-04-21

**Authors:** Roshni Basu, Yuedan Chen, Emily J. Quann, Morgan Huse

**Affiliations:** Immunology Program, Memorial Sloan-Kettering Cancer Center, New York, New York, United States of America; University of Iowa, United States of America

## Abstract

The immunological synapse (IS) formed between a T cell and its cognate antigen-presenting cell (APC) enables the directional secretion of cytolytic and inflammatory molecules. Synaptic architecture is established in part by a two-step cascade of novel protein kinase C (nPKC) isozymes. PKCε and PKCη arrive at the IS first, and occupy the entire synaptic membrane. Then, PKCθ accumulates in a smaller zone at the center of the contact. We investigated the molecular basis for this differential recruitment behavior using chimeric nPKC constructs and total internal reflection fluorescence microscopy. Our studies revealed that the V3 linker just N-terminal to the kinase domain plays a crucial role in specifying nPKC localization. Substitution of this linker switched the scope and the kinetics of PKCθ accumulation to that of PKCε and PKCη, and vice versa. Although the V3 was necessary for synaptic compartmentalization, it was not sufficient, as the tandem C1 domains were also required to mediate membrane association. Together, these results suggest a model whereby the V3 linker controls nPKC sub-compartmentalization after initial C1 domain-mediated accumulation at the IS.

## Introduction

Recognition of cognate peptide major histocompatibility complex (pMHC) by a T cell on the surface of an antigen-presenting cell (APC) induces the formation of a specialized cell-cell junction known as an immunological synapse (IS). The IS mediates adhesion, regulates intracellular signaling, and enables polarized secretion of cytokines and cytolytic factors toward the APC [1]. Imaging studies of the mature IS have, in general, revealed a radially symmetric structure containing distinct concentric domains. The T cell antigen receptor (TCR), which recognizes pMHC, accumulates at the very center of the contact. This cluster of TCR is surrounded by an adhesion ring containing the α_L_β_2_ integrin LFA-1, which is, in turn, surrounded by a ring of filamentous actin (F-actin) at the outer edge of the IS. These three domains are sometimes referred to as supramolecular activation clusters (SMACs): the TCR cluster is called the central SMAC (cSMAC), the LFA-1 accumulation the peripheral SMAC (pSMAC), and the F-actin ring the distal SMAC (dSMAC). Studies suggest that the relative placement of key signaling proteins within this structural framework can profoundly affect T cell function [2]–[4]. Our understanding of the organizational logic guiding compartmentalization within the IS, however, remains incomplete.

The novel protein kinase C (nPKC) subfamily, which includes PKCθ, PKCε, PKCη, and PKCδ, drives gene transcription and effector responses downstream of the TCR [5], [6]. Three out of four of these isoforms (PKCθ, PKCε, and PKCη) accumulate at the IS, where they guide the polarization of the microtubule cytoskeleton [7]. Interestingly, the recruitment behavior exhibited by these proteins is nonuniform. PKCε and PKCη, which are more closely related to each other than to any other PKC isozymes, arrive at the IS first and occupy the entire interface. By contrast, PKCθ accumulates ∼5 seconds later and is contained within the peripheral F-actin ring (i.e. it occupies the cSMAC and pSMAC only) [7]. The molecular basis for these differences is not known and is the subject of this study.

All nPKCs have the same basic structure comprising an N-terminal C2 domain, a set of tandem C1 domains, and a C-terminal serine/threonine kinase domain [8]. It is generally thought that the C1 domains, which bind to the lipid second messenger diacylglycerol (DAG), play a central role in recruiting nPKCs to the IS. TCR engagement induces marked accumulation of DAG in the synaptic membrane, and studies suggest that the C1 domains of PKCθ, PKCε, and PKCη can recognize this DAG and respond accordingly [7], [9]–[11]. Nevertheless, there are indications that the C1 domains, on their own, are insufficient for sustained accumulation and subcompartmentalization within the IS, and that other elements within the nPKC structure, including the kinase domain and the C2 domain, are also critical for the process [12]–[14]. Of particular relevance to this study, it was recently shown that the V3 linker, a poorly conserved sequence that connects the tandem C1 and kinase domains, is required for PKCθ localization to the cSMAC [15]. Whether the V3 linkers of other nPKCs have similar functions, however, is not known.

Here, we have examined the molecular basis for differential nPKC compartmentalization at the IS. We show that while the tandem C1 domains are required for IS accumulation, it is the V3 linker that specifies both the kinetics and the spatial scope of recruitment. These results demonstrate how localization is encoded within nPKC structure and provide insight into the patterning of cell-cell interfaces.

## Results

### The V3 hinge region determines synaptic nPKC localization

**Figure 1:**
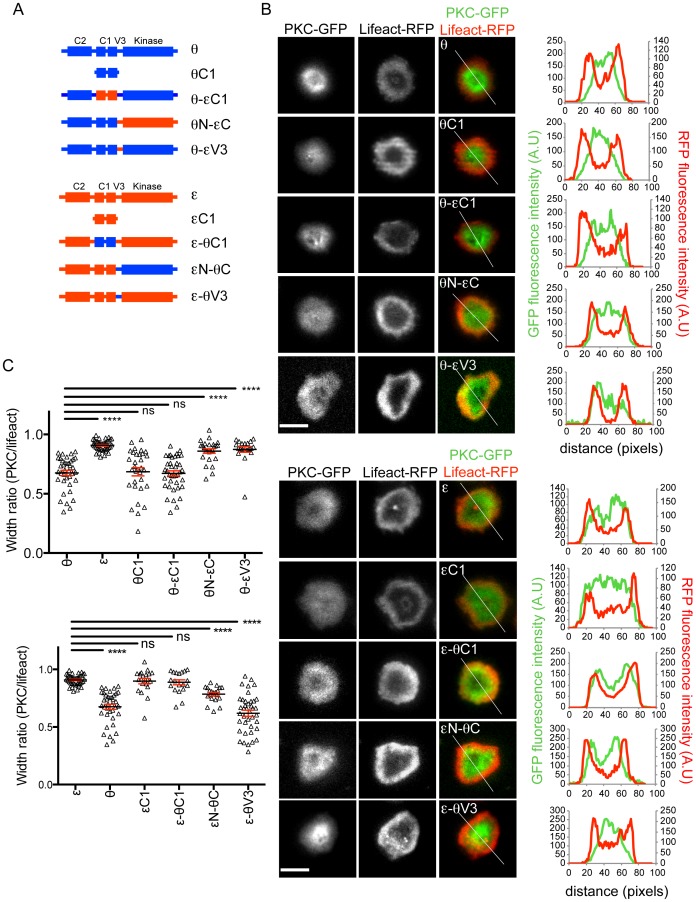
The V3 linker determines localization of PKCε and PKCθ. 5C.C7 T cells were transduced with the indicated nPKC constructs and Lifeact-RFP, stimulated on bilayers containing pMHC and ICAM-1, fixed, and imaged by TIRF microscopy. (A) Schematic diagrams of the chimeric constructs used in this study. (B) Representative images are shown to the left (scale bars = 10 µm), with linescans (derived from the white lines shown in the rightmost images) depicting the radial fluorescence intensity of nPKCs and Lifeact to the right. (C) Quantification of the width ratio for each PKC construct (see Materials and Methods), with black lines and red error bars denoting mean and s.e.m., respectively (n≥20 cells). **** indicates p<0.0001, ns indicates not significant. Data were pooled from at least 2 independent experiments for each construct.

To identify the regions of PKCθ, PKCε, and PKCη that specify their distinct accumulation patterns, we prepared a panel of chimeric constructs in which domains from PKCε or PKCη were introduced into PKCθ, and vice versa (Fig. 1A; Fig. S1). These constructs were GFP-labeled and transduced into primary CD4^+^ T cells expressing the 5C.C7 TCR, which recognizes the moth cytochrome c_88-103_ peptide bound to the class II MHC molecule I-E^k^. The cells were then stimulated on supported lipid bilayers containing cognate peptide-MHC (pMHC) and ICAM-1, a ligand for LFA-1. On surfaces of this kind, T cells form radially symmetric synapses bounded by a peripheral ring of F-actin [16]–[18]. Consistent with previous observations [7], total internal reflection fluorescence (TIRF) imaging revealed that full-length PKCθ localized within the F-actin ring, while PKCε and PKCη distributed over the entire synaptic membrane (Fig. 1B; Fig. S1A). Indeed, PKCε and PKCη often appeared to accumulate preferentially in a more peripheral IS zone than PKCθ, roughly corresponding to the pSMAC/dSMAC boundary. GFP alone gave a rather diffuse and uniform signal in these experiments (Fig. S1B), indicating that the patterns observed for the nPKC isoforms reflected active compartmentalization within the IS.

To quantify the relative size of synaptic nPKC accumulations, we calculated the ratio between the diameter of the PKC distribution and the diameter of the F-actin ring. As expected, this “width ratio” was approximately one for PKCεand PKCη, and significantly less than one for PKCθ (Fig. 1C; Fig. S1C). Interestingly, constructs containing only the tandem C1 domains of PKCθ and PKCε (θC1 and εC1) largely recapitulated the localization patterns of their respective full-length proteins (Fig. 1B, C). Although the boundaries of θC1 accumulation were less well defined than those of intact PKCθ, this did not significantly increase the quantifiable diameter of the accumulation pattern.

These initial experiments hinted that the C1 domains were responsible for the differential recruitment of PKCθ and PKCε. If this were the case, then replacing this region of PKCθ with that of PKCε, and vice versa, should be sufficient to switch their localization. Surprisingly, however, the recruitment pattern of PKCθ bearing the C1 domains of PKCε (θ-εC1) was essentially indistinguishable from that of full length PKCθ (Fig. 1B, C). Furthermore, constructs containing the PKCθ C1 domains in the context of either PKCε (ε-θC1) or PKCη (η-θC1) behaved identically to full length PKCε and PKCη, respectively (Fig. 1B, C; Fig. S1A, C). Hence, the tandem C1 domains were insufficient to specify recruitment when transferred into a different nPKC.

These results implied that the actual localization motif must reside elsewhere. Accordingly, we analyzed chimeras containing the N-terminal regulatory domains (C2 and tandem C1) of either PKCθ or PKCε fused to the C-terminal portion of the other protein (θN-εC and εN-θC), which contained the kinase domain and V3 hinge. Interestingly, εN-θC localized within the actin ring, similar to full length PKCθ, while θN-εC distributed uniformly over the IS (Fig. 1B, C). Thus, the C-terminal half of each construct dictated its localization. Next, we assessed the importance of the V3 linker using PKCε and PKCη constructs containing the V3 of PKCθ (ε-θV3 and η-θV3) and a PKCθ construct containing the V3 region of PKCε (θ-εV3). Strikingly, ε-θV3 and η-θV3 displayed the centralized localization pattern of full length PKCθ, while θ-εV3 mirrored the broad distribution of PKCε and PKCη (Fig. 1B, C; Fig. S1A, C). Collectively, these data indicate that the V3 linker is the primary structural determinant controlling nPKC compartmentalization within the IS. Although the isolated tandem C1 domains recapitulate the localization of their respective full-length proteins, the V3 can modulate and even override this behavior.

### The V3 hinge region controls the kinetics of nPKC recruitment

We next examined whether the V3 linker might also influence the kinetics of nPKC recruitment. For this purpose, we utilized a TCR photoactivation and imaging assay based on a caged form of the MCC-I-E^k^ complex that is nonstimulatory until irradiated with UV light [19]. 5C.C7 T cells are attached to coverslips coated with this caged pMHC. Then, individual cells are stimulated by brief UV irradiation of micron-scale regions beneath them. PKCε and PKCη are recruited to the irradiated region in ∼90 seconds, with PKCθ following ∼5 seconds later [7].

**Figure 2:**
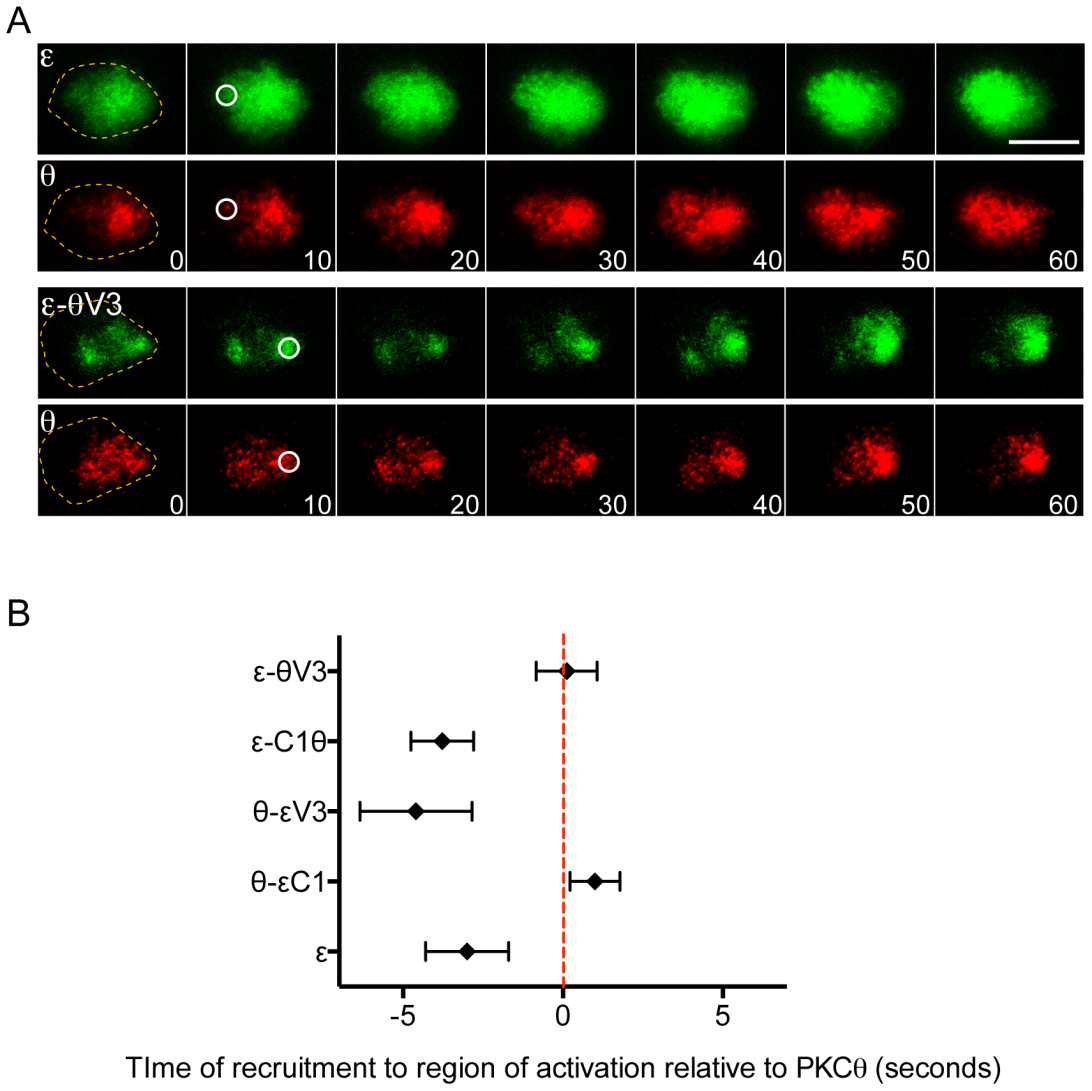
The V3 linker dictates the kinetics of PKCε and PKCθ recruitment. 5C.C7 T cells expressing the indicated GFP-labeled nPKC chimeras together with PKCθ-RFP were imaged by TIRF microscopy and UV irradiated on surfaces containing photoactivatable pMHC. (A) Representative time-lapse montages (time shown in seconds at lower right corner), with the time and region of UV irradiation indicated by white circles. Dotted lines in the first image denote the extent of the T cell membrane on the glass. Scale bar = 10 µm. (B) Offset times separating the recruitment of nPKC chimeras from the recruitment of PKCθ, calculated by cross-correlation analysis of at least 10 paired responses. Error bars = s.e.m. Data are representative of at least two independent experiments.

To assess the relative recruitment kinetics of nPKC chimeras, we analyzed T cells expressing GFP-labeled forms of each construct together with RFP-labeled PKCθ. The temporal offset between the arrival of the GFP-labeled protein and the recruitment of PKCθ was then determined by cross-correlation analysis [9]. Consistent with prior results [7], we found that full-length PKCε and PKCη preceded PKCθ to the irradiated region by 3.0±1.3 seconds and 2.5±0.63 seconds, respectively (Fig. 2A, B; Fig. S2). Swapping of the tandem C1 domains between PKCθ and either PKCε or PKCη did little to change this temporal pattern. θ-εC1 recruitment was essentially coincident with that of PKCθ, while ε-θC1 and η-θC1 arrived earlier (Fig. 2B; Fig. S2). Substitution of the V3 linker, however, induced a striking reversal. θ-εV3 behaved like wild type PKCε and PKCη, preceding wild type PKCθ to the irradiated region by 4.6±1.8 seconds (Fig. 2B). Conversely, ε-θV3 and η-θV3 exhibited little to no temporal offset with PKCθ (Fig. 2A, B; Fig. S2), indicating that the presence of θV3 effectively delays IS recruitment. Thus, the V3 linker controls not only the spatial but also the temporal pattern of nPKC compartmentalization at the IS.

### The V3 hinge region, while necessary, is insufficient for synaptic compartmentalization

**Figure 3:**
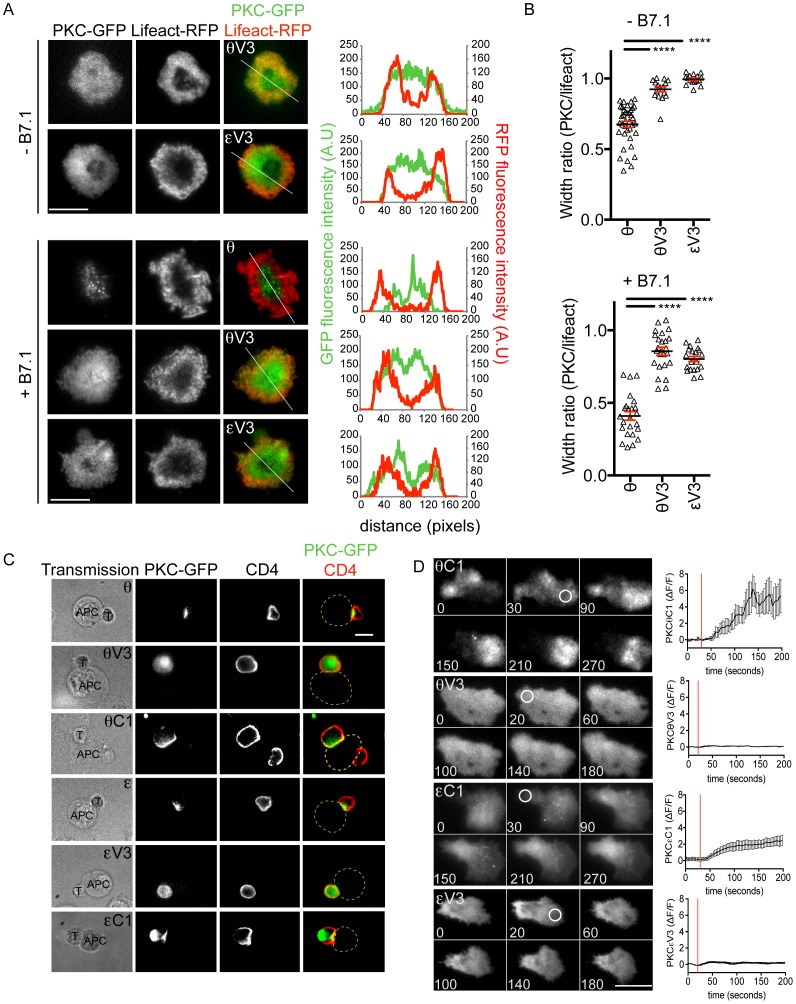
The V3 region is insufficient for recruitment to the IS. (A–B) 5C.C7 T cells expressing the indicated GFP-labeled nPKC constructs and Lifeact-RFP were stimulated on bilayers containing pMHC, ICAM-1, and B7.1 as indicated, and then imaged by TIRF microscopy. (A) Representative images are shown to the left (scale bar = 10 µm), with linescans (derived from the white lines shown in the rightmost images) depicting the radial fluorescence intensity of nPKCs and Lifeact to the right. (B) Quantification of the width ratio for each PKC construct (n≥20 cells), with black lines and red error bars denoting mean and s.e.m., respectively. **** indicates p<0.0001 (C) 5C.C7 T cells expressing the indicated GFP-labeled nPKC constructs were mixed with CH12 APCs pre-loaded with MCC peptide, fixed, and stained with an anti-CD4 antibody (to label T cells). Images of T cell-APC conjugates (representative of >20 conjugates) are shown, with T cells and APCs indicated in the transmission images. Yellow dotted lines in the images on the right denote the edge of the APC. (D) 5C.C7 T cells expressing the indicated GFP-labeled nPKC constructs were imaged by TIRF microscopy and UV irradiated on surfaces containing photoactivatable pMHC. Left, representative time-lapse montages (time indicated in seconds at lower right corner) with the time and region of UV irradiation indicated by white circles. Right, background corrected accumulation (ΔF/F) in the irradiated region (n = 6 per graph), with red lines indicating UV irradiation and error bars denoting s.e.m. Data are representative of at least two independent experiments.

The results described above suggested that the V3 linker might be sufficient to specify IS compartmentalization. To test this hypothesis, we used TIRF microscopy to assess the synaptic localization patterns of isolated θV3 and εV3, which we expressed in 5C.C7 T cells as GFP fusions. Neither construct recapitulated the constrained accumulation of full-length PKCθ; instead, they exhibited uniform distributions over the entire interface (Fig. 3A, B). To confirm these observations, we characterized θV3 and εV3 localization by epifluorescence microscopy in conjugates formed by 5C.C7 T cells and antigen-loaded CH12 B cells. Both constructs distributed evenly throughout the T cell, with no obvious preference for the T cell-APC interface (Fig. 3C; Fig. S3). Conversely, full-length PKCθ and PKCε localized to the IS in an antigen dependent manner (Fig. 3C; Fig. S3), consistent with previous studies. θC1 and εC1 also accumulated synaptically in these experiments, although a significant amount of εC1 did remain in a cytoplasmic pool (Fig. 3C; Fig. S3).

To further characterize the localization behavior of θV3 and εV3, we examined their recruitment dynamics in TCR photoactivation experiments. Neither construct exhibited accumulation to the irradiated region, whereas θC1 and εC1 both polarized in a manner similar to full-length PKCθ and PKCε, respectively (Fig. 3D; Fig. 2A). Taken together, these results indicate that the V3 linker is insufficient for nPKC compartmentalization within the IS, and suggest that DAG recognition by the C1 domains plays an important role in the process.

Both θV3 and εV3 were expressed at the expected molecular weight (Fig. S4), indicating that the absence of synaptic localization that we observed could not be attributed to degradation or premature termination of the constructs. We also assessed whether engagement of the costimulatory receptor CD28 was required for V3-dependent effects, as it had been shown previously that the PKCθ V3 linker could form a ternary complex with CD28 and the Src-family kinase Lck [15]. Stimulation of 5C.C7 T cells on bilayers containing pMHC, ICAM-1, and the CD28 ligand B7.1 induced the recruitment of full-length PKCθ into centrally located microclusters (Figure 3A, B). Consistent with previous reports [3], the formation of these microclusters was strictly dependent on the presence of B7.1. Neither θV3 nor εV3, however, formed microclusters in this manner (Fig. 3A, B). Indeed, the localization patterns exhibited by these constructs were essentially insensitive to CD28 costimulation. Collectively, our data indicate that the V3 linker, although necessary, is insufficient for nPKC compartmentalization at the IS.

## Discussion

Although nPKC recruitment is a hallmark of IS formation, the factors controlling the localization behavior of these proteins have remained largely unresolved. In this study, we demonstrated that the V3 linker plays a central and instructive role by specifying the scope of nPKC compartmentalization within the IS. θV3 constrains recruitment to the cSMAC and pSMAC, while εV3 and ηV3 promote accumulation over the entire synaptic membrane. These results explain the differential localization of PKCε, PKCη, and PKCθ, and provide insight into the molecular mechanisms that guide IS formation.

In our hands, the isolated nPKC V3 regions could not specify IS localization on their own. Rather, they appeared to function together with other components, most likely the DAG binding C1 domains. These data contrast with a recent report indicating that the V3 is both necessary and sufficient for synaptic localization of PKCθ [15]. Although it is difficult to explain this discrepancy at present, it is worth noting that the previous study imaged human θV3, whereas we used a mouse construct. There are some notable differences between the human and mouse proteins near the C-terminal end of the linker that could conceivably enable human θV3 to accumulate synaptically in the absence of other domains. Further studies are required to resolve this matter. Nevertheless, any potential the V3 regions might have to localize to the IS on their own does not appear to be required for controlling compartmentalization in the context of a full-length nPKC.

Taken together with previous work [7], [10], [12], [15], our data are consistent with a cooperative mechanism for synaptic nPKC recruitment involving both the V3 hinge and the tandem C1 domains. One could imagine an ordered process in which DAG binding by the C1 domains attaches nPKCs to the synaptic membrane, after which localization is further refined by the V3. Within this framework, V3 dependent compartmentalization of PKCθ at the T cell IS could be mediated by interactions with Lck and CD28, as previously described [15]. Our results, however, indicate that θV3 can influence localization in the absence of CD28 engagement, implying the existence of other binding partners or an alternative localization mechanism.

In that regard, it tempting to speculate that V3 regions might control synaptic compartmentalization by modulating the activity of the tandem C1 domains. DAG is known to form a plasma membrane gradient that peaks at the center of the IS [9], [10]. By altering the affinity of the C1 domains for DAG, the V3 could potentially affect both the kinetics and the scope of nPKC accumulation. Intramolecular interactions between θV3 and the C1 domains could reduce DAG binding, resulting in delayed recruitment to a smaller synaptic region. Conversely, interactions mediated by εV3 or ηV3 could enhance affinity, leading to faster recruitment over the entire IS. Consistent with this model, it has been shown that full-length PKCθ binds to DAG and phorbol esters with lower affinity than does PKCε [20], [21]. It remains to be seen, however, whether the V3 regions play any role in this discrepancy.

It is important to note that our data do not exclude a role for other nPKC domains in additional aspects of the recruitment response, in particular for sustained accumulation at the IS. It was recently shown, for example, that the kinase activity of PKCθ plays an important role in synaptic retention, presumably by phosphorylating key substrates [12]. In addition, the C2 domain has been implicated in the coalescence of membrane-localized PKCθ into signaling microclusters [13]. Indeed, it is quite possible that the structural determinants of synaptic nPKC accumulation change over time as the lipid and protein composition of the IS evolve.

In conclusion, we have defined the V3 linker as a key determinant working in conjunction with the tandem C1 domains to control nPKC localization. Recent studies have highlighted the importance of lipid second messengers like DAG and PIP_3_ for organizing proteins within the IS [7], [9], [17]. By combining with or functionally modifying the lipid binding activities of adjacent domains, motifs like the V3 hinge can further refine synaptic structure and, by extension, T cell function.

## Materials and Methods

### Ethics statement

The animal protocols used for this study were approved by the Institutional Animal Care and Use Committee of Memorial Sloan-Kettering Cancer Center.

### Constructs

PKC chimeras were constructed using a 3-step PCR stitching protocol where three PKC fragments were amplified separately and used as templates in subsequent PCR steps. Fragments encoding full-length chimeras were digested with XhoI/Not1I and ligated into the MSCV (murine stem cell virus) retroviral expression vector upstream of and in-frame with GFP. The following chimeras were created: θ-εC1 (a.a. 160–281 of PKCθ replaced by 170–292 of PKCε), θN-εC (a.a. 282–707 of PKCθ replaced by 293–737 of PKCε), θ-εV3 (a.a. 282–383 of PKCθ replaced by 293–411 of PKCε), ε-θC1 (a.a. 170–292 of PKCε replaced by 160–281 of PKCθ), εN-θC (a.a. 293–737 of PKCε replaced by 282–707 of PKCθ), ε-θV3 (a.a. 293–411 of PKCε replaced by 282–383 of PKCθ), η-θC1 (a.a. 172–295 of PKCη replaced by 160–281 of PKCθ); η-θV3 (a.a. 296–346 of PKCη replaced by 282–383 of PKCθ). θC1 and εC1 encompassed a.a. 160–281 of PKCθ and 170–292 of PKCε, respectively.

### Cells

T cell blasts were prepared by mixing lymphocytes from 5C.C7 TCR transgenic mice with irradiated splenocytes from B10A mice at a ratio of 1∶5 with 5 µM MCC peptide. Cells were cultured in RPMI medium containing 10% (vol/vol) FCS. IL-2 (30 IU/ml) was added 18 h after lymphocyte isolation, and cells were split as needed in RPMI medium containing IL-2 (30 IU/ml). At day 3 after primary stimulation, cells were co-infected with retroviruses containing GFP tagged PKC constructs and Lifeact-RFP as previously described [9]. CH12 cells were cultured in RPMI medium containing 10% (vol/vol) FCS.

### Imaging

An inverted fluorescence video microscope (Olympus IX-81) equipped with Slidebook software (Intelligent Imaging Innovations) was used for image acquisition. For imaging of the IS, cells were fixed in 2% paraformaldehyde 10 minutes after activation on supported lipid bilayers coated with streptavidin and then biotinylated MCC–I-E^k^ (0.5 µg/ml) and biotinylated ICAM-1 (1 µg/ml) [17]. TIRF images of fluorescently labeled T cells in contact with these bilayers were acquired with a 60× or 150× objective lens (NA 1.45; Olympus). Photoactivation experiments were performed on photoactivatable glass surfaces prepared as described [9]. A Mosaic digital diaphragm (Photonic Instruments) attached to a mercury lamp (Olympus) was used for UV irradiation. TIRF images were captured every 2 s for 5 min with a 150× objective lens (NA 1.45; Olympus) using 488-nm and 561-nm lasers (Melles Griot) for imaging of GFP and RFP, respectively. Cells were photoactivated for 1 s or 1.5 s during the tenth time point of the capture series. T cell-APC conjugates were prepared by mixing CH12 cells (preincubated in the presence or absence of 5 µM MCC peptide) with 5C.C7 T cells for 10 minutes at 37°C, followed by fixation on polylysine-coated coverslips. Fixed conjugates were stained with an anti-CD4 antibody (clone GK1.5; UCSF monoclonal antibody core) to visualize the T cells and then imaged using a 60× objective lens (NA 1.45; Olympus).

Images were analyzed using SlideBook, MATLAB (MathWorks), and Prism (GraphPad) software. The GFP and RFP signals in each cell were directly compared by calculation of the ratio of the distribution widths for the two probes (width ratio) averaged along two perpendicular lines drawn across the cell. Recruitment of signaling probes in response to TCR stimulation was analyzed by plotting of mean fluorescence intensity at the photoactivated region versus time after background correction. Cross-correlation analysis was applied as described [9] using normalized fluorescence intensity curves from paired GFP and RFP channels.

## Supporting Information

Figure S1 **The V3 linker determines localization of PKCη.** 5C.C7 T cells were transduced with the indicated GFP-labeled constructs and Lifeact-RFP, stimulated on bilayers containing pMHC and ICAM-1, fixed, and imaged by TIRF microscopy. (A and B) Representative images are shown to the left (scale bar = 10 µm), with linescans to the right (derived from the white lines shown in the images) depicting the radial fluorescence intensity of Lifeact and either nPKC constructs (A) or GFP alone (B). (C) Quantification of the width ratio for each PKC construct (see Materials and Methods), with black lines and red error bars denoting mean and s.e.m., respectively (n≥50 cells). **** indicates p<0.0001, ns indicates not significant. Data were pooled from at least 2 independent experiments for each construct.
(TIF)

Figure S2 **The V3 linker dictates the kinetics of PKCη recruitment.** 5C.C7 T cells expressing the indicated GFP-labeled nPKC chimeras together with PKCθ-RFP were imaged by TIRF microscopy and UV irradiated on surfaces containing photoactivatable pMHC. Offset times separating the recruitment of nPKC chimeras from the recruitment of PKCθ were calculated by cross-correlation analysis of at least 10 paired responses. Error bars = s.e.m. Data are representative of at least two independent experiments.
(TIF)

Figure S3 **Localization of nPKC constructs in conjugates formed in the absence of cognate peptide.** 5C.C7 T cells expressing the indicated GFP-labeled nPKC constructs were mixed with CH12 APCs not preloaded with cognate peptide, fixed, and stained with anti-CD4 antibodies (to label T cells). Images of T cell-APC conjugates (representative of >20 conjugates) are shown, with T cells and APCs indicated in the transmission images. Yellow dotted lines in the images on the right denote the edge of the APC.
(TIF)

Figure S4 **Expression of θV3 and εV3 in T cells.** 5C.C7 T cells expressing GFP-labeled θV3 or εV3 were lysed and analyzed by Western blot using an antibody against GFP. Data are representative of 2 independent experiments.
(TIF)
