# Supplementary figures and images for: The Variable Hinge Region of Novel PKCs Determines Localization to Distinct Regions of the Immunological Synapse
Source: PLoS One. 2014 Apr 21;9(4):e95531. doi: 10.1371/journal.pone.0095531 (PMC3994095; doi:10.1371/journal.pone.0095531)

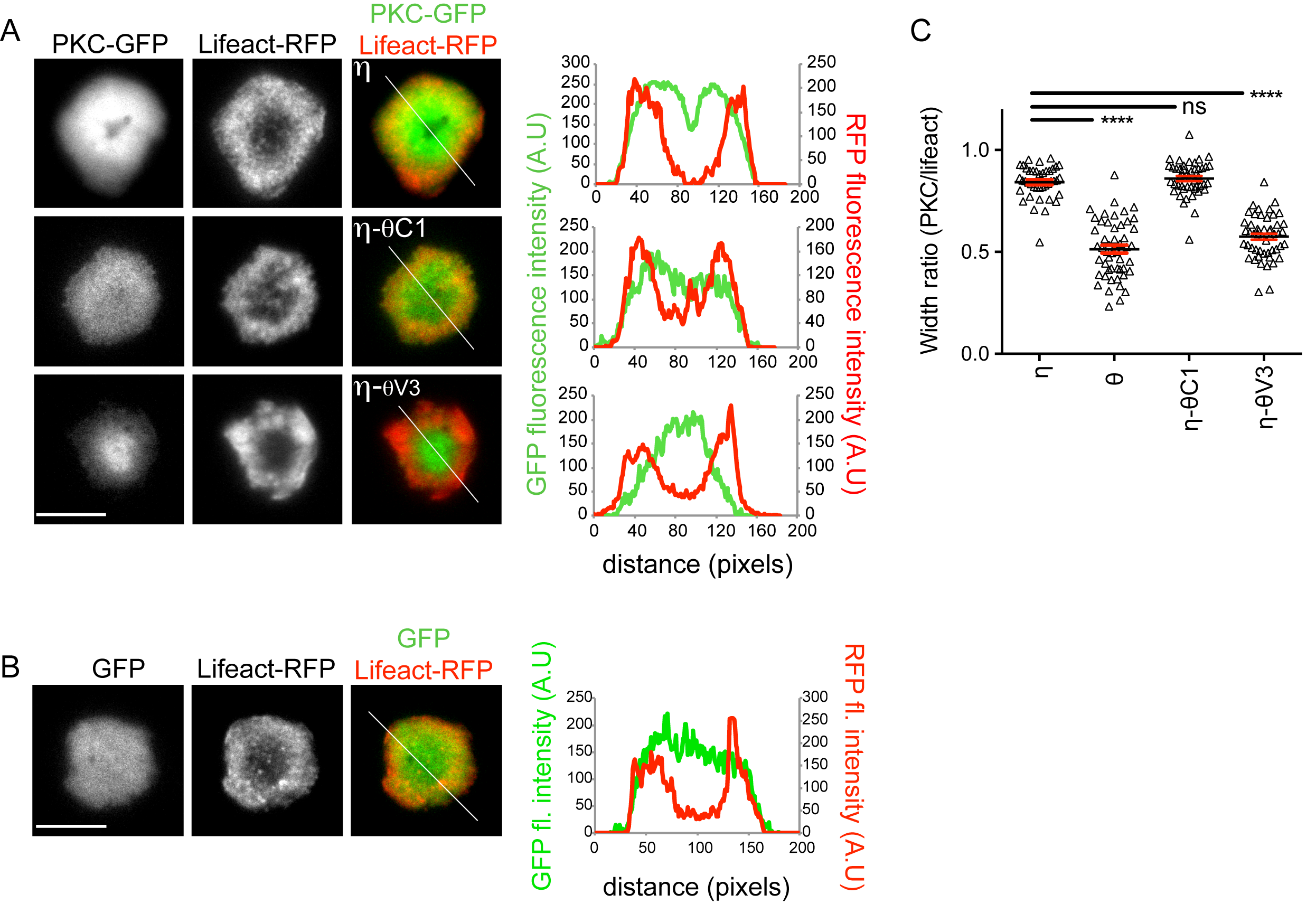

Supplement: Figure S1 — The V3 linker determines localization of PKCη. 5C.C7 T cells were transduced with the indicated GFP-labeled constructs and Lifeact-RFP, stimulated on bilayers containing pMHC and ICAM-1, fixed, and imaged by TIRF microscopy. (A and B) Representative images are shown to the left (scale bar = 10 µm), with linescans to the right (derived from the white lines shown in the images) depicting the radial fluorescence intensity of Lifeact and either nPKC constructs (A) or GFP alone (B). (C) Quantification of the width ratio for each PKC construct (see Materials and Methods), with black lines and red error bars denoting mean and s.e.m., respectively (n≥50 cells). **** indicates p<0.0001, ns indicates not significant. Data were pooled from at least 2 independent experiments for each construct. (TIF) [file pone.0095531.s001.tif]

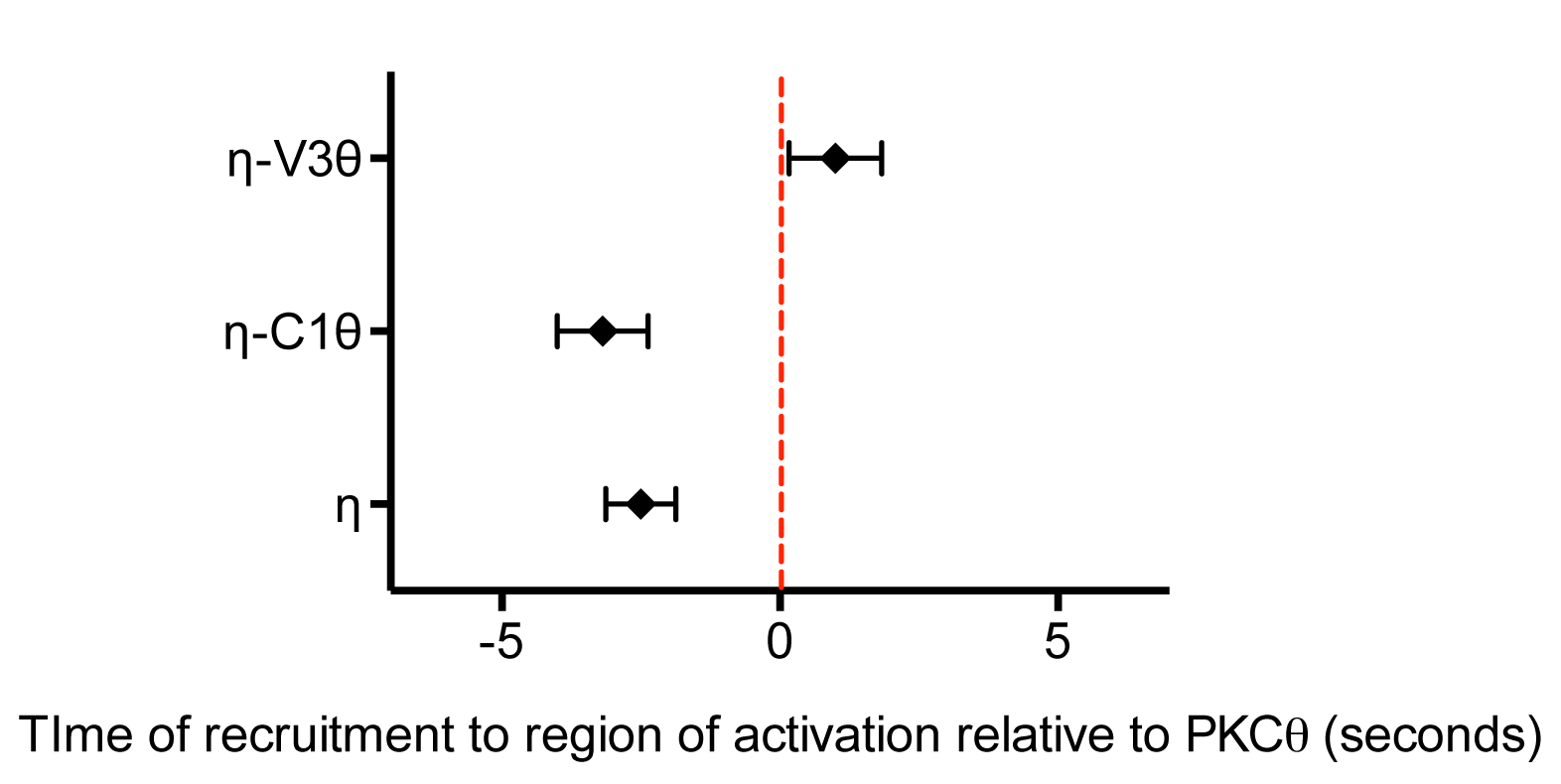

Supplement: Figure S2 — The V3 linker dictates the kinetics of PKCη recruitment. 5C.C7 T cells expressing the indicated GFP-labeled nPKC chimeras together with PKCθ-RFP were imaged by TIRF microscopy and UV irradiated on surfaces containing photoactivatable pMHC. Offset times separating the recruitment of nPKC chimeras from the recruitment of PKCθ were calculated by cross-correlation analysis of at least 10 paired responses. Error bars = s.e.m. Data are representative of at least two independent experiments. (TIF) [file pone.0095531.s002.tif]

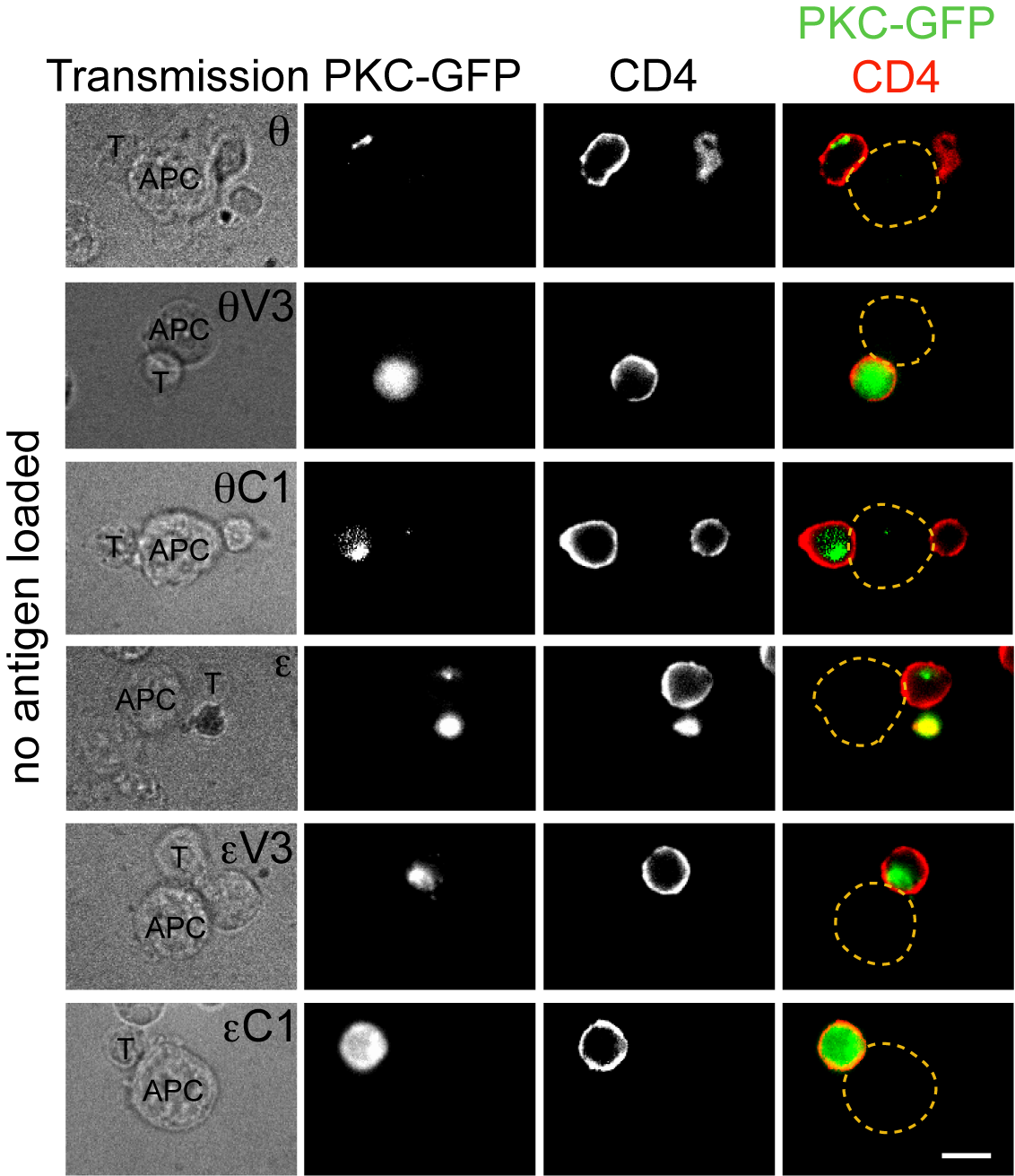

Supplement: Figure S3 — Localization of nPKC constructs in conjugates formed in the absence of cognate peptide. 5C.C7 T cells expressing the indicated GFP-labeled nPKC constructs were mixed with CH12 APCs not preloaded with cognate peptide, fixed, and stained with anti-CD4 antibodies (to label T cells). Images of T cell-APC conjugates (representative of >20 conjugates) are shown, with T cells and APCs indicated in the transmission images. Yellow dotted lines in the images on the right denote the edge of the APC. (TIF) [file pone.0095531.s003.tif]

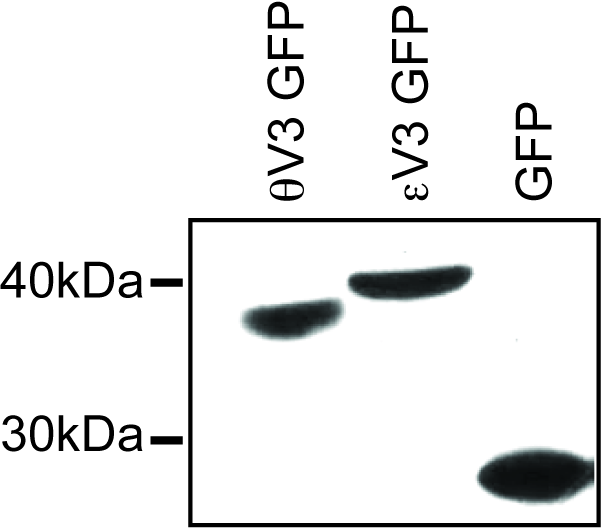

Supplement: Figure S4 — Expression of θV3 and εV3 in T cells. 5C.C7 T cells expressing GFP-labeled θV3 or εV3 were lysed and analyzed by Western blot using an antibody against GFP. Data are representative of 2 independent experiments. (TIF) [file pone.0095531.s004.tif]
